# FA2H Exhibits Tumor Suppressive Roles on Breast Cancers via Cancer Stemness Control

**DOI:** 10.3389/fonc.2019.01089

**Published:** 2019-10-24

**Authors:** Xiaofeng Dai, Shuo Zhang, Hongye Cheng, Dongyan Cai, Xiao Chen, Zhaohui Huang

**Affiliations:** ^1^Wuxi School of Medicine, JiangNan University, Wuxi, China; ^2^School of Biotechnology, Jiangnan University, Wuxi, China; ^3^Wuxi Cancer Institute, Affiliated Hospital of Jiangnan University, Wuxi, China

**Keywords:** cancer stem cell, triple negative breast cancer, FA2H, tumor suppressive role, STAT3/IL6

## Abstract

**Background:** Triple negative breast cancers are aggressive, enriched with cancer stem cells, and lack effective targeted therapies with little side effects.

**Methods:** We isolated cancer stem cells from two triple negative breast cancer cell lines via cell sorting following transcriptome sequencing, bioinformatics analysis, experimental and clinical validations, as well as functional investigations to explore genes capturing triple negative breast cancer features for improved diagnosis and therapeutics in clinics.

**Results:** We found that FA2H is under-expressed in triple negative breast cancers both *in vitro* and in clinics, and FA2H suppresses cancer stemness via inhibiting the STAT3/IL6 axis and NFkB signaling.

**Conclusions:** This study reports the tumor suppressive roles of FA2H on breast cancer cells through cancer stemness control. FA2H and other candidates unveiled in this study that capture the features of cancer stem cells may contribute as diagnostic marker and/or effective therapeutic targets for improved triple negative breast cancer management.

## Introduction

Despite the considerable contributions of traditional diagnostic and treatment strategies, such as screening mammography and adjuvant therapy, on breast cancer death control, it still remains the leading cause of women mortality worldwide (1, 2). Breast cancer is not a single disease but comprised of various subtypes with distinct patient pathological features and treatment responses (3–7). It can be roughly grouped as luminal (luminal A and B), HER2 positive, and triple negative breast cancers (TNBC) according to the presence or expression of estrogen receptor (ER), progesterone receptor (PR), and epidermal growth factor receptor 2 (HER2). Successes on targeted therapies have been reported on luminal tumors such as Tamoxifen (8), and HER2 positive cancer such as Herceptin (9). TNBCs are characterized by a poor prognosis and lack of effective targeted therapies due to the absence of the three primary surface receptors (10–13). Classical treatments on TNBCs rely on anthracycline- and/or taxane-based chemotherapies, which suffer from unavoidable adverse effects (14, 15). Efforts on TNBC specific agents primarily fall into two regimes, i.e., angiogenesis inhibitors and PARP inhibitors. However, the efficacy of anti-angiogenic drugs on breast cancer treatment may not seem to be fully justified given the unacceptable toxicity (16–18), and PARP inhibitors rest on the blockage of the alternative PARP-dependent DNA repair pathway and only benefit BRCA mutation-associated hereditary breast cancers that overlap with TNBCs but not represent it as a whole (19). Therefore, identifying the molecular features of TNBCs is of vital importance for the establishment of effective treatment strategies of such cancers and the associated diagnosis.

Cancer stem cells (CSCs) have been proposed to empower cancer cells with self-renew capacity and resistance toward drug treatment (20–23). They comprise a small tumor cell subpopulation that is self-renewable, drive tumorigenesis and contribute to tumor cellular heterogeneity (23, 24). The consistencies between many properties of CSCs and TNBCs have shed light on the discovery of effective TNBC treatment strategies. We are motivated to find genes capturing the features of TNBCs by interrupting CSC signaling driving the aggressiveness of such tumors.

By isolating CSCs (represented as CD44^+^/CD24^−/low^ cohort) from TNBC cells following transcriptome sequencing and a sequential bioinformatics analysis as well as clinical association studies, we selected 8 genes for experimental validation. Evidences at both transcriptional and translational levels as well as functional studies using established stable cells with *FA2H* modulation suggested the tumor suppressive roles of *FA2H* on cancer stemness and cell migration via inhibiting the STAT3/IL6 axis and NFkB mediated signaling. Taken together, we propose the tumor suppressive roles of *FA2H* on TNBC control and the driving mechanism, which may potentially be used in the therapeutic design against TNBCs.

## Materials and Methods

### Cell Lines

Twelve breast cancer cell lines (purchased from OBIOER Biosciences Co. LTD), including two luminal A, two luminal B, two HER2 positive, and six triple negative cell lines were used in this study and cultured under conditions as recommended (Table S1). Most of these cell lines come from American Type Culture Collection (ATCC) (1), except for three triple negative cell lines (SUM149PT, SUM159PT, SUM1315MO2) obtained from the collections of Dr. S. Ethier's laboratory (2).

### Exploration of Candidate Genes

#### Flow Cytometry Analysis and Cell Sorting

Subconfluent cells were washed once with phosphate-buffered saline (PBS) and harvested with trypsin. Detached cells were washed once and resuspended at 107 cells/ml in PBS with 1% FBS (wash buffer). One hundred microliter cell suspension was added into Round-Bottom tube (BD Falcon), and cells were stained with CD24-PE (20 μl, BD Pharmingen) and CD44-APC antibodies (20 μl, BD Pharmingen) or their respective isotype controls at 4°C in the darkness for 30 min. The labeled cells were washed and fixed in the wash buffer. The CD44+/CD24–/low and non- CD44+/CD24–/low cell percentage, representing the proportion of cancer stem cells (CSCs) and non-CSCs, were analyzed using FACS Caliber flow cytometer (FACS) (BD Biosciences) and isolated by BD FACS Aria II(Becton Dickinson) within 1 h after staining. Flow cytometry analysis was conducted three times when assessing cancer stem cell percentage, with student *T*-test p ≤ 0.05 being considered statistically significant.

#### Transcriptome Sequencing and Bioinformatics Analysis

The CSC and non-CSC cohorts were isolated from SUM149PT and HCC1937 given their balanced percentage of CSC and non-CSC percentages (40–60% CSC in each cell line) using cytometry sorting. Total RNA of CSCs and non-CSCs of SUM149PT and HCC1937 as well as total RNA of SUM159PT (considered as CSCs, CD44+CD24– cells accounts for 96.1 ± 3.30%) were extracted. RNA concentration and quality were determined by Nanodrop 2000 (Thermo) and Agilent 2100 Bioanalyzer (Agilent). A total of 50 ng RNA was sequenced by HiSeqTM 2500 (Illumina) in Oebiotech Company.

Genes differentially expressed between CSCs and non-CSCs in both SUM149PT and HCC1937 were checked among the expression profiles of SUM159PT which is comprised primarily of CSCs. Genes over- and under-expressed in CSCs of SUM149PT and HCC1937 and fall into the upper and lower 25% quantiles of SUM159PT were selected as candidates, with the false discovery rate (FDR) ≤ 0.0001 and enrichment score ≥ 1 set as the cut off threshold for gene selection. The “ebayes” function from the “limma” R package was used to explore differentially expressed genes.

### Validation of Candidate Genes

#### Quantitative Real-Time PCR

Total RNA was extracted from all 12 breast cancer cell lines (TIANGEN, Beijing, China), followed by RNA reverse transcription (TaKaRa, Dalian, China), with the output cDNA concentration being 10 ng/μl. Primers designed for candidate genes and used for quantitative reverse transcription polymerase chain reaction (qRT-PCR) validation (ordered from GENEWIZ) were listed in Table S2. The qRT-PCR was performed using the Quantifast SYBR Green PCR kit (QPK-201, Toyobo) with the ABI PRISM 7500 Quantitative PCR system (Life Technologies, Carlsbad, CA, USA). Each sample was examined in triplicates and the PCR products were normalized by the expression of GAPDH, the internal control. Statistical significance was assessed using student *T*-test with the cutoff being set at *p* ≤ 0.05.

#### Western Blotting

Total proteins of all cells were extracted using RIPA Lysis BufferRIPA Lysis Buffer (Beyotime, China) supplemented with protease and phosphatase inhibitor cocktails (Selleck, USA). Protein concentrations were quantified by BCA (Beyotime, China). Thirty microgram total protein was applied to run on a 12% SDS-PAGE gel, followed by transferation onto polyvinylidene di?uoride membranes. The membranes were blocked using 5% fat free milk or 5% BSA for 1 h and then incubated with primary antibodies for 2 h at room temperature. FA2H antibody (proteintech), IL6 (proteintech), STAT3 (proteintech), Caspase 7 (Cell Signaling Technology) ERK (Cell Signaling Technology), JNK (Cell Signaling Technology), and NF-kB (Cell Signaling Technology) were diluted by 1:600. The p-STAT3 (Santa Cruz), p-NFkB (Cell Signaling Technology), p-JNK (Cell Signaling Technology) and p-ERK (Cell Signaling Technology) were diluted by 1:300. GAPDH (1:2,500, proteintech) was used as an internal control. HRP-conjugated anti-rabbit IgG and anti-mouse IgG was used at a dilution rate of 1:4,000 (biosharp) and incubated for 1 h at the room temperature, following by washing using Tris-buffered saline with Tween three times for 5 min each. Immunoblotting signals were detected using the Western blotting detection system (OmegaLumG).

Nuclear proteins were extracted using Nuclear and Cytoplasmic Protein Extraction Kit (Beyotime, China).

### Functional Studies of the Candidate Gene

#### Stable Cell Line Establishment With Up and Down Gene Regulation

SKBR3 and MDAMB231 cells were selected to establish stable cell lines with FA2H down- and up-regulation for functional studies, as FA2H is highly and lowly expressed in both lines, respectively.

The FA2H sequence was synthesized and subcloned into the lentiviral expression vector pLenti-EF1a-EGFP-P2A-Puro-CMV-3Flag and the pLKD-CMV-G&PR-U6-shFA2H lentiviral plasmid, respectively, by OBiO (China).

Breast cancer cells were transfected with lentifection using the lentifectin transfection reagent (Gene Pharma). Cells were incubated 24 h following the addition of appropriate amount of virus suspension. Total mRNA and total protein were detected 48–72 h later.

#### Gene Silencing

SKBR3 cells were plated in the 6-well plate, and siRNAs (forward strand: 5′-GCUAUUACCUCAUCAUGCUTT-3′, backward strand: 5′-AGCAUGAUGAGGUAAUAGCTG-3′) (Table S3) were synthesized from Gene Pharma (Suzhou, China) to silence FA2H. SKBR3 cells were transfected with FA2H siRNA and non-targeting siRNA (forward strand: 5′-UUCUCCGAACGUGUCACGUdTdT-3′, backward strand: 5′-ACGUGACACGUUCGGAGAAdTdT-3′) separately and in combination using siRNA mate transfection agent (Gene Pharma, Suzhou, China). The siRNA concentration used for transfection was 50 nM. The cells were incubated in 5% CO_2_ at 37°C for 33 and 57 h, respectively, before testing the silencing effect at transcriptional and translation levels using qRT-PCR and Western blot. Triplicates were conducted, with statistical significance cutoff being set at *p* ≤ 0.05 from student *T*-test.

#### Cell Proliferation

Cell viability was assessed by a Cell Counting Kit 8 (CCK-8, Japan) according to the manufacturer's protocol. Cells were incubated for 24 h, following the addition of 5 μl CCK8 in each well. After 2 h incubation, the absorbance value was detected using microplate spectrophotometer.

#### Transwell

Transwell assays were performed to test cell migration. Cells were incubated for 48 h under normoxic and anaerobic conditions, respectively. Cell medium was added to the lower layer of 24 well-culture plate and the chambers were placed in the medium. Cells were collected following pancreatic digestion, re-suspended and added to the chambers (2 × 10^5^/well). The culture media inside the chambers were discarded after 20 h, and cells were washed by PBS. Migrated cells under the chambers were fixed by methanol followed by staining with 0.1% crystal violet solution.

#### Tumorsphere Formation

Single cell was plated in ultralow attachment 6-well plates (Corning) at 25,000 cells per well for primary tumorsphere formation. After incubation for 7 days, mammospheres were collected and dissociated by trypsin. As the secondary tumorsphere formation, 200 cells per well were plated in ultralow attachment 96-well plates. Cells were grown in StemXVivo Serum-Free Media (R&D) containing 2 U/ml Heparin (Tocris) and 0.8 μg/ml Hydrocortisone (Tocris). Mammospheres were harvested 7 days later, and tumorsphere was calculated under inverted phase-contrast light microscope (Olympus). The experiment was repeated three times. Statistical difference on secondary tumorsphere formation was assessed using student *T*-test with *p* ≤ 0.05 as the significance criterion.

When the effect of IL6 on tumorsphere formation was examined, recombinant human IL6 (Peprotech) was supplemented at concentration of 0, 100, 200 ng/μl in the beginning of the primary and secondary tumorsphere formation stages.

#### Cancer Stem Cell Percentage

Alterations on CD44+CD24–/low subset cells were examined using FACS (BD Biosciences). The experiment was repeated three times. The statistical significance was determined using student *T*-test, with *p* ≤ 0.05 being set as the cutoff.

### Clinical Sample Collection, IHC Staining, and Statistical Analysis

A total of 70 human primary breast cancer tissues were collected at Affiliated Hospital of Jiangnan University from August to October in 2017 with written informed consent (Table 1), and this project was approved by the Clinical Research Ethics Committees of Affiliated Hospital of Jiangnan University.

**Table 1:** Phenotypic information of 70 collected samples.

**Features**	***N***
**Subtypes**	
Luminal	43
HER2+	9
TNBC	18
**Ages (years)**	
<54	35
≥54	35
**Stage**	
I + II	21
III + IV	49
**Histology**	
Invasive ductal carcinoma	56
Invasive lobular carcinoma	1
Ductal carcinoma *in situ*	6
Mucious carcinoma	3
Mucious carcioma *in situ*	3
Cyst	1

IHC staining was performed on 4 μm sections from formalin fixed paraffin embedded breast cancer tissues using anti-FA2H antibody (1:500, Proteintech). IHC staining of FA2H was observed in the cytoplasm of tumor cells, and a scale of 0 to 3 was used to score relative expression intensity. Chi-squared test for trend in proportions was conducted to examine the significance of FA2H IHC status in differentiating breast cancer subtypes using R, where scores 0 and 1 were considered as low FA2H expression and 2 and 3 were grouped as high FA2H expression.

## Results

### Exploration of Candidate Genes

#### Transcriptome Sequencing Reveals Candidate Genes Capturing Cancer Stemness

We identified 414, and 832 genes differentially expressed between CSCs and non-CSCs from SUM149PT and HCC1937 cell lines, respectively, with 112 overlapping genes regulated in the same direction in both lines (Tables S4, S5). Among the 112 genes, 64 were down-regulated in CSCs of SUM149PT and HCC1937 and ranked in the lower 25% quantile of SUM159PT, and 16 were up-regulated in CSCs of SUM149PT and HCC1937 and ranked in the upper 25% quantile of SUM159PT (Table S4). Kaplan Meier Plotter (25) (http://kmplot.com/analysis/index.php?p=service&cancer=breast), a database containing clinical information and gene expression data on 3,951 breast cancer patients, shows significant (*p* ≤ 0.05) prognostic value on breast cancer survival for 8 out of the 80 genes (Figure S1). These eight genes (*FA2H, ALOX15B, IL2RG, TNNC1, KCNMA1, SERPINE1, TGFBR2, TNC*) constitute the candidate gene panel for experimental validation (Table 2).

**Table 2:** Candidate genes differentially expressed between stem and non-stem cell subpopulations.

**Gene name**	**SUM149PT**		**HCC1937**		**Regulatory direction on CSCs**	**SUM159PT quantile**	**Kaplan-Meier Plotter**
	**log_**2**_FC**	***p*-value**	**log_**2**_FC**	***p*-value**			***p*-value**
ALOX15B	−7.38	1.28E-27	−2.03	3.17E-19	Down	<25%	3.50E-06
FA2H	−2.53	2.00E-16	−1.08	5.30E-20	Down	<25%	3.40E-03
IL2RG	−2.58	8.59E-04	−1.7	4.77E-11	Down	<25%	1.10E-05
TNNC1	−2.57	6.07E-03	−2.07	9.37E-07	Down	<25%	9.20E-06
KCNMA1	3.3	1.30E-136	2.03	1.25E-15	Up	>75%	5.60E-09
SERPINE1	3.36	0.00E+00	1.8	2.70E-133	Up	>75%	1.30E-03
TGFBR2	2.27	1.80E-284	1.18	4.40E-47	Up	>75%	3.90E-05
TNC	2.77	0.00E+00	1.38	9.76E-49	Up	>75%	9.50E-04

*Log_2_FC and p-value are short for log_2_ fold change and p-values in SUM149PT and HCC1937 cells, respectively; SUM159PT quantile shows the quantile each gene is located among the expression of all genes in SUM159PT*.

#### Experimental Validations Reveal FA2H as a Candidate Gene With Tumor Suppressive Roles

**Figure 1:**
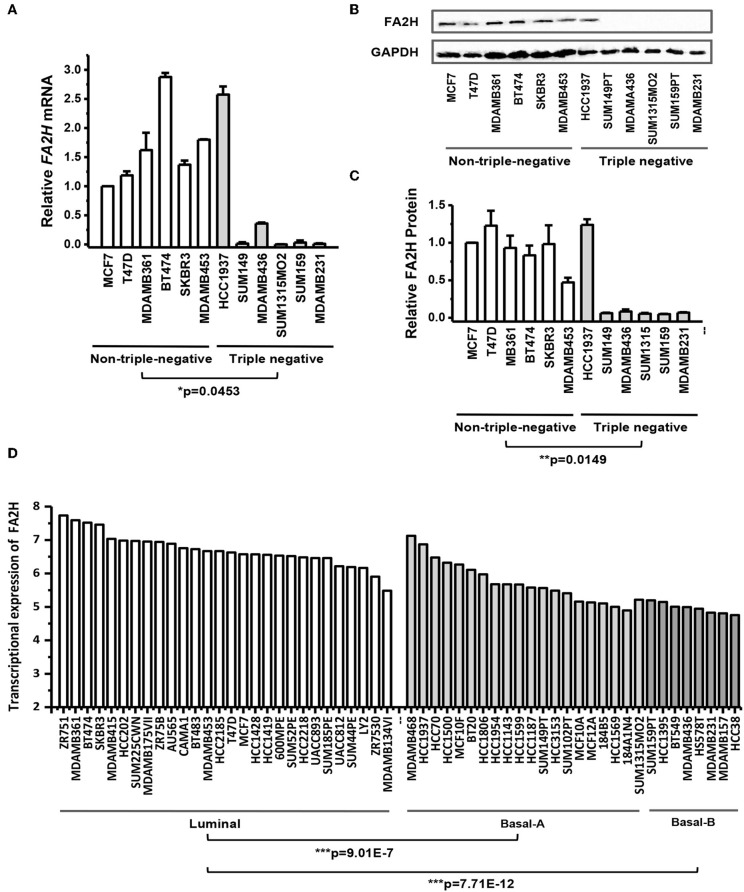
FA2H expression among breast cancer cell lines. **(A)** *FA2H* gene expression among different breast cancer cell lines by qRT-PCR. **(B)** FA2H protein expression among different breast cancer cell lines by Western-blotting. **(C)** FA2H protein expression quantified using the ImageJ software. **(D)** *FA2H* gene expression across 56 breast cancer cell lines (26).

Both qRT-PCR (Figure 1A) and western blotting (Figures 1B,C) results showed that FA2H could clearly distinguish the 12 breast cancer cell lines while the rest candidate genes could not (Figure S2). Except for HCC1937 which harbors a PTEN deletion, FA2H was lowly expressed in triple negative cell lines (Figure 1A, *p* = 0.045). The same results were confirmed by western blot (Figure 1B) and quantified using ImageJ software (Figure 1C, *p* = 0.015). By exempting HCC1937 from the analysis, the *p*-values comparing TNBC and non-TNBC further decrease to 6.9E-4 at the transcriptional level and 3.81E-5 at the translational level. We analyzed *FA2H* expression across 56 cell lines (26) (https://www.ebi.ac.uk/arrayexpress/experiments/E-MTAB-181/) and found that it could significantly distinguish cell lines of different subtypes, with the highest expression observed in luminal cells following basal-A cells (including HCC1937) and the lowest expression obtained in the basal-B cells (*p* = 9.01E-7 for luminal vs. basal-A and *p* = 7.71E-12 for luminal vs. basal-B, Figure 1D).

#### Clinical Sample Validations Reveal FA2H as a Candidate Gene With Tumor Suppressive Roles

**Figure 2:**
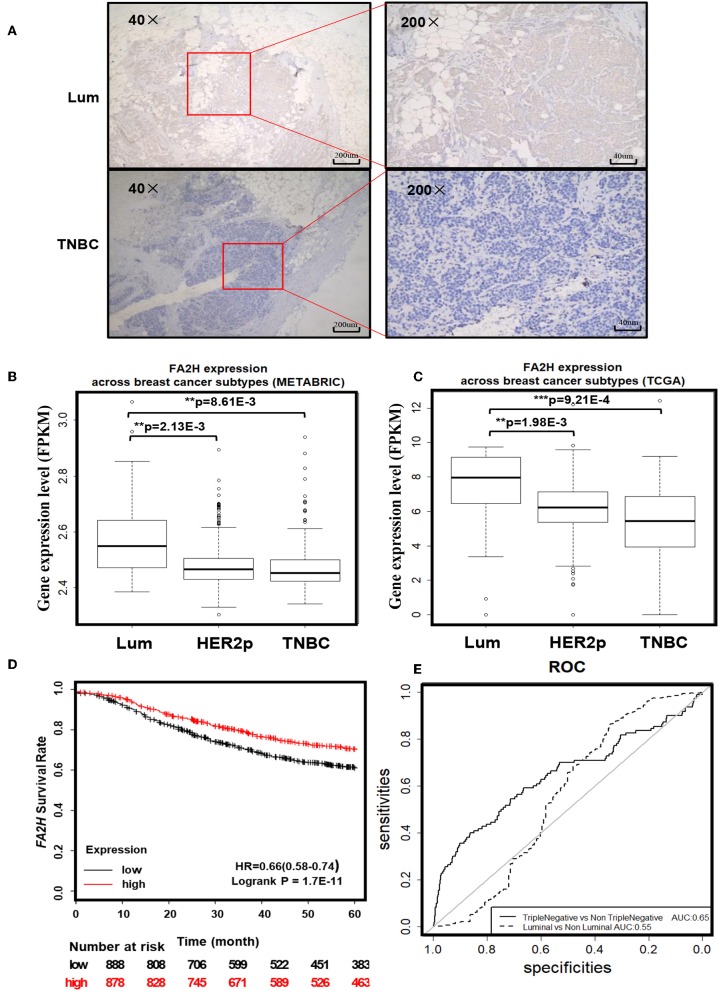
Clinical evidence suggesting potential tumor suppressive roles of FA2H. **(A)** IHC staining of FA2H between triple negative and luminal breast tumors. *FA2H* mRNA expression stratified by breast cancer subtypes using **(B)** METABRIC data and **(C)** TCGA data. **(D)** Kaplan–Meier survival curves evaluating the association of *FA2H* (probe is 234963_s_at) expression with 5 years' patient survival in breast cancer patients using KM plotter. **(E)** Precision and recall curves showing the potential diagnostic value of FA2H in identifying triple negative breast cancers using TCGA data.

IHC staining of 70 collected breast cancer samples (Table 1) suggest significant differences in FA2H expression among patients of different subtypes (*p* = 0.003, Figure 2A, Table 3), and FA2H expression is significantly associated with the molecular features of luminal and TNBCs (*p* = 0.0012 and 0.0268 for luminal and TNBCs, respectively, Table 3). Similar results were obtained using METABRIC and TCGA data, where FA2H expression was slightly lower in the TNBC subtype (Figures 2B,C). Gene expression association analysis using Kaplan-Meier plotter reveals a significant protective effect of FA2H on breast cancer patient 5 years' survival (Figure 2D, *p* = 1.7E-11, HR = 0.66). The precision and recall curve drawn from TCGA data shows that FA2H expression could potentially be used to characterize triple negative tumors (Figure 2E, AUC = 0.65).

**Table 3:** Immunohistochemistry staining results of FA2H and chi-squared test for trend in proportions.

**Subtype**	**FA2H: 0-1**	**FA2H: 2-3**	**Total**	***p*-value**
Luminal	20	23	43	0.0012
HER2+	8	1	9	0.0698
TNBC	15	3	18	0.0268
Total	43	27	70	
*p*-value			0.0031	

*FA2H:0–1 represents samples with FA2H expression scored as 0 or 1, FA2H:2–3 shows samples with FA2H expression scored as 2 or 3. P values were computed from chi-squared test for trends in proportions using R*.

### Functionality Studies on *FA2H*

#### Establishment of Stable Cell Lines With FA2H Up- and Down-Regulations

**Figure 3:**
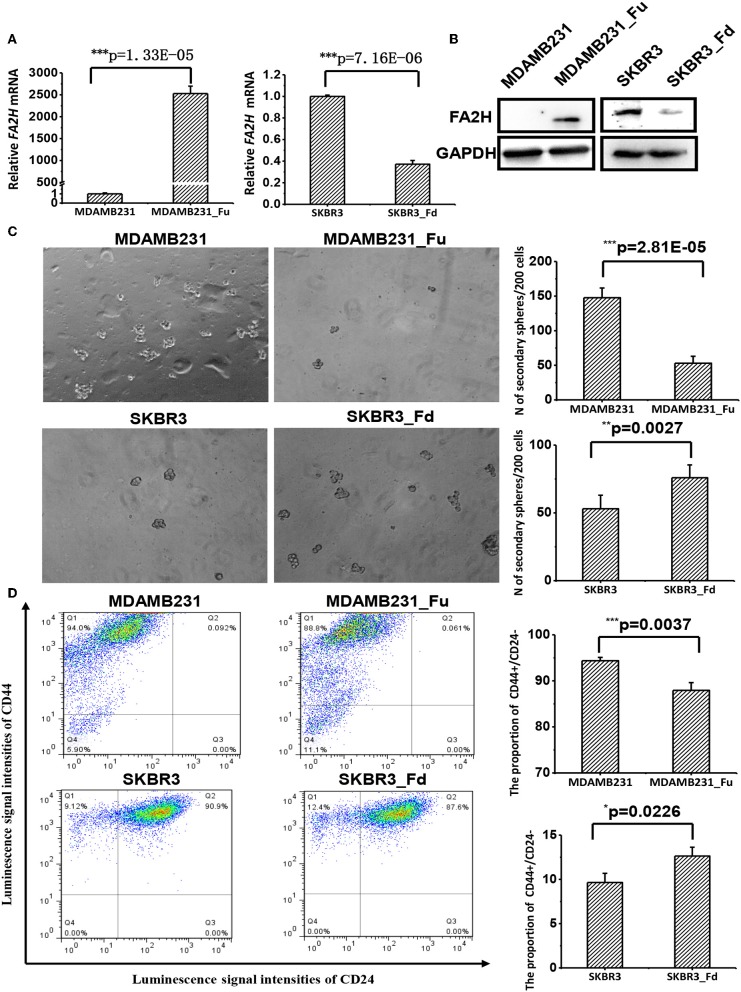
The effects of up- and down-regulating *FA2H* on breast cancer stemness. **(A)** FA2H expression in SKBR3_Fd, SKBR3, MDAMB231_Fu, MDAMB231 cells at the gene expression level. **(B)** FA2H expression in SKBR3_Fd, SKBR3, MDAMB231_Fu, MDAMB231 cells at the protein expression level. **(C)** Tumorsphere formation of MDAMB231_Fu, MDAMB231, SKBR3_Fd and SKBR3 cells. **(D)** Cancer stem cell percentage in MDAMB231_Fu, MDAMB231, SKBR3_Fd, and SKBR3 cells according to flow cytometry analysis.

We established a stable MDAMB231 cell line over-expressing FA2H, named MDAMB231-Fu, and a stable SKBR3 cell line with low FA2H expression, namely SKBR3-Fd, using lentiviral transfection to study the functionalities of FA2H. FA2H was up-regulated 2,825-folds (*p* = 1.33E-05, Figure 3A) in MDAMB231-Fu, and down-regulated 2.69 times (*p* = 7.16E-06, Figure 3A) in SKBR3-Fd at the transcriptional level. In the western blotting, clear band of FA2H was observed in MDAMB231-Fu cells whereas no band was seen in MDAMB231 cells; and visible down-regulation of FA2H was confirmed in SKBR3-Fd as compared with SKBR3 (Figure 3B). MDAMB231 and SKBR3 cells were used to model CSCs and non-CSCs given their extremely imbalanced CSC percentage (i.e., 96.1 ± 3.3% for MDAMB231, and 0.58 ± 0.03% on CD44+CD24– percentage). We did not use CSCs and non-CSCs isolated from triple negative cells in the functional studies as it is difficult to obtain sufficient CSC cells for all subsequent experiments and guarantee CSC purity during cell cultivation. It was worth mentioning that any triple negative cells with low FA2H expression and high CSC percentage such as SUM159PT, MDAMB436, SUM1315MO2 besides MDAMB231 can be used for constructing stable cells over-expressing *FA2H*, and any non-triple negative cells with high *FA2H* expression and low CSC percentage such as MCF7, T47D, MDAMB361, BT474, MDAMB453 besides SKBR3 can be used for establishing stable cells under-expressing *FA2H* in the functional studies, which form an integrate part independent from cell lines selected for FA2H identification.

#### FA2H Suppresses Cancer Cell Stemness

MDAMB231-Fu exhibited reduced self-renew ability than MDAMB231 with statistical significance (*p* = 2.81E-05, Figure 3C), and showed a lower percentage of cancer stem cell (*p* = 0.0037, Figure 3D). SKBR3-Fd cells, on the other hand, showed significantly increased self-renew ability than SKBR3 (*p* = 0.0027, Figure 3C), and elevated level of cancer stem cell percentage (*p* = 0.0226, Figure 3D).

#### FA2H Suppresses Cell Migration and Invasion

**Figure 4:**
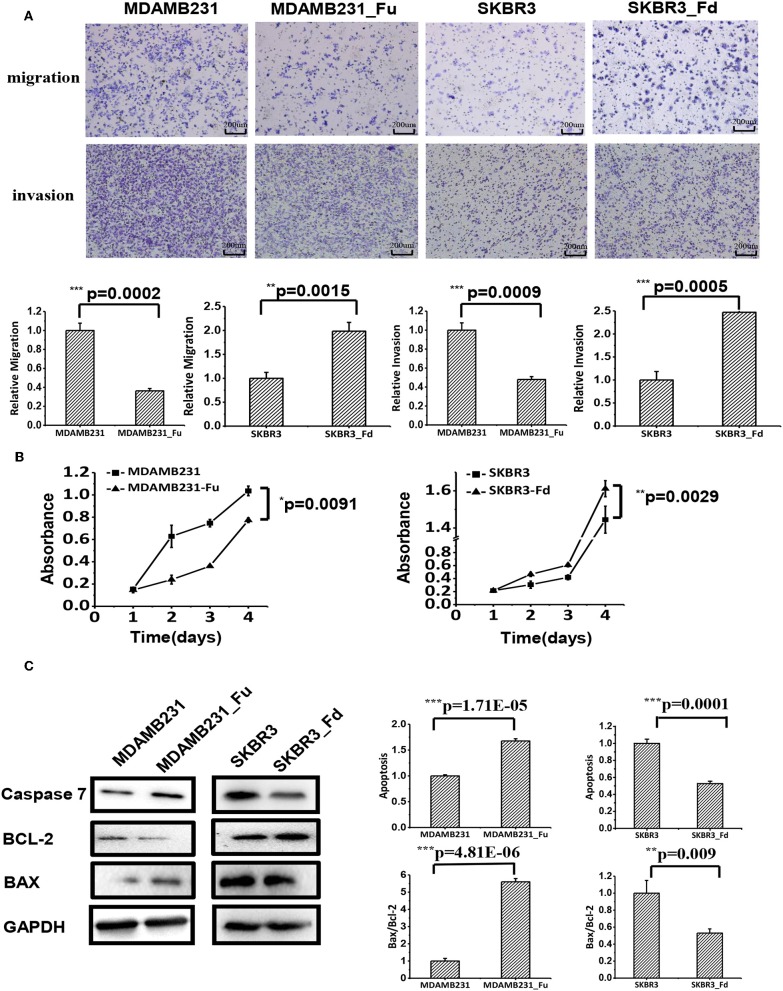
The effects of up- and down-regulating *FA2H* on cell migration, growth and apoptosis of breast cancer cells. **(A)** Cell migration of MDAMB231_Fu, MDAMB231, SKBR3_Fd, SKBR3 cells as tested using transwell. **(B)** The growth of SKBR3_Fd, SKBR3, MDAMB231_Fu, MDAMB231 cells. **(C)** Apoptosis of SKBR3_Fd, SKBR3, MDAMB231_Fu, MDAMB231 cells as tested on Caspase 7 (apoptosis executioner) expression using Western-blot.

Cell migration and invasion were significantly deterred in MDAMB231-Fu as compared with MDAMB231 cells (*p* = 0.0002 for migration, *p* = 0.0009 for invasion, Figure 4A), and enhanced with statistical significance in SKBR3-Fd than SKBR3 cells (*p* = 0.0015 for migration, *p* = 0.0005 for invasion) as measured by the transwell assay (Figure 4A).

#### FA2H Inhibits Cancer Cell Growth

MDAMB231-Fu grows slower than MDAMB231 with statistical significance (*p* = 0.0091, Figure 4B), and SKBR3-Fd grows significantly faster than that of SKBR3 (*p* = 0.0029, Figure 4B).

#### FA2H Promotes Cancer Cell Apoptosis

The apoptosis of MDAMB231-Fu was examined using Caspase 7 [an executioner of cell apoptosis (27)], Bax, Bcl-2. Caspase 7 expression and Bax/Bcl-2 ratio were significantly higher in MDAMB231_Fu than MDAMB231 cells (p = 1.71E-05 for Caspase 7, *p* = 4.81E-06 for Bax/Bcl-2, Figure 4C), and were significantly lower in SKBR3-Fd than SKBR3 cells (*p* = 0.0001 for Caspase 7, *p* = 0.009 for Bax/Bcl-2, Figure 4C).

#### FA2H Inhibits the STAT3/IL6 Axis That Stimulates Cancer Stemness

**Figure 5:**
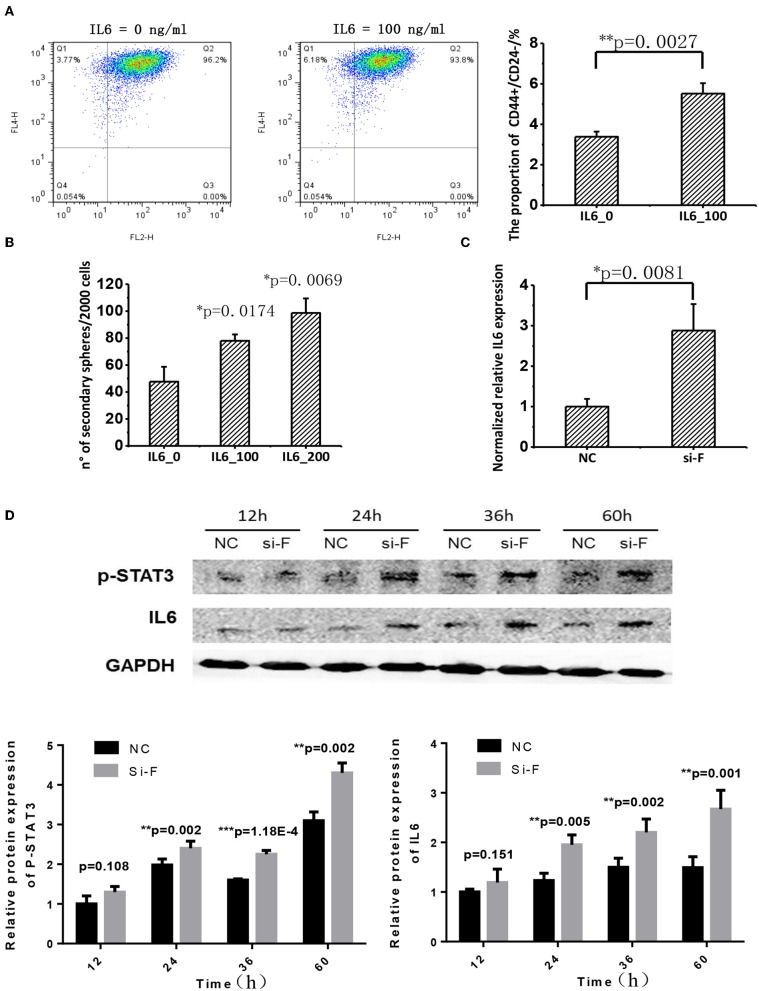
The effect of FA2H on STAT3/IL6 and the effect of IL6 on cancer stemness. **(A)** Comparisons on CSC percentage after adding IL6 at 100 ng/ml. **(B)** Comparisons on tumorsphere formation after adding IL6 at 100 and 200 ng/ml. **(C)** Silencing efficiency and the effect of silencing *FA2H* on *IL6* gene expression at 36 h after transfection. **(D)** IL6 expression and STAT3 phosphorylation at Tyr705 after silencing *FA2H* at different time points.

**Figure 6:**
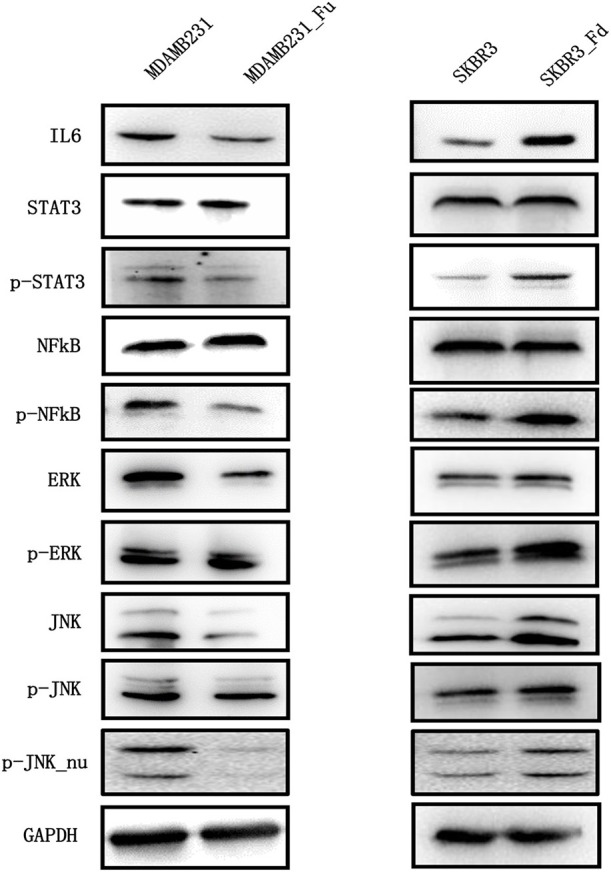
The expression of key proteins of the IL6/STAT3, NFkB, MAPK/ERK, MAPK/JNK pathways in breast cancer cells before and after modulating *FA2H* expression. “Fd” and “Fu” each represents down- and up-ward gene expression regulation, “*p*” means phorsphoralation.

IL6 is a cytokine implicated in inflammation induction and CSC maintenance (28–30). Supplementing cells with 100 ng/ml IL6 can significantly enhance CD44+CD24–/low proportion in SKBR3 cells (Figure 5A). IL6 addition significantly promoted SKBR3 tumorsphere formation and the effect increases with IL6 concentration, i.e., tumorsphere increases to 1.5-folds (*p* = 0.0174) and 2-folds (*p* = 0.0069), respectively, when IL6 is 100 and 200 ng/ml, respectively (Figure 5B). Silencing FA2H using siRNA increases IL6 gene expression as measured at 36 h (*p* = 0.0081, Figure 5C). We measured the protein level of IL6 expression and STAT3 phosphorylation at 12, 24, 36, and 60 h after transfection, and observed increasingly up-regulated IL6 expression and STAT3 phosphorylation levels with the statistical significance being reached starting from 24 h (*p* = 0.005, 0.002, 0.001 at 24, 36, 60 h for IL6; *p* = 0.002, 1.18E-4, 0.002 at 24, 36, 60 h for STAT3 phosphorylation, Figure 5D). Examination on IL6 expression as well as the total and phosphorylation levels of STAT3 expression between MDAMB231_Fu and MDAMB231 and those between SKBR3_Fd and SKBR3 confirms the repressed level of IL6 and STAT3 phosphorylation in MDAMB231_Fu and elevated level in SKBR3_Fd (*p* = 0.0009 for IL6 and *p* = 5.34E-05 for STAT3 phosphorylation in MDAMB231_Fu, *p* = 3.78E-06 for IL6 and *p* = 4.55E-05 for STAT3 phosphorylation in SKBR3_Fd, Figure 6, Figure S3).

#### FA2H Suppresses NFkB Phosphorylation but Does Not Alter the Phosphorylation Level of JNK and ERK

Reduced phosphorylation but not total protein level of NFkB was observed in MDAMB231-Fu cells as compared with MDAMB231 cells (*p* = 9.38E-06 for NFkB phosphorylation, Figure 6, Figure S3), and increased phosphorylation level of NFkB was shown in SKBR3-Fd as compared with SKBR3 without significant alteration on total NFkB protein level (*p* = 0.0001 for NFkB phosphorylation, Figure 6, Figure S3).

FA2H significantly reduced the phosphorylation (Thr202/Tyr204) and total protein levels of ERK in MDAMB231-Fu cells (*p* = 8.52E-06 for ERK and *p* = 0.002 for p-ERK, Figure 6, Figure S4), and up-regulated those in SKBR3-Fd cells (*p* = 0.0003 for ERK and *p* = 0.001 for p-ERK, Figure 6, Figure S4), as compared with their corresponding original cells, respectively. FA2H did not alter the phosphorylation level of JNK (Thr183/Tyr185) but decreased its total protein and nuclear phosphorylation levels in MDAMB231-Fu cells (*p* = 1.22E-6 for JNK, *p* = 0.0006 for p-JNK_nu, Figure 6, Figure S5), and vice versa as compared with each original cell line accordingly (*p* = 1.33E-5 for JNK and *p* = 0.0033 for p-JNK_nu, Figure 6, Figure S5).

## Discussion

Three triple negative cell lines, SUM149PT, HCC1937, and SUM159PT, were used for the identification of CSC feature genes that are representative of triple negative tumor cells, due to the evenly distributed CSC and non-CSC subpopulations in the first two cell lines as required for FACS and the representativeness of SUM159PT for the rest triple negative cell lines considered full of CSCs.

We initially found eight candidates capturing CSC properties. After transcriptional and translational *in vitro* validations, FA2H was revealed having potential tumor suppressive roles, which is likely under-expressed in triple negative breast tumors (Figures 1A–C, *p* = 0.0453). *FA2H* encodes a fatty acid 2-hydroxylase which catalyzes the initial step of straight chain fatty acid α-oxidation (31, 32). It was reported to promote cell differentiation and remarkably increased during 3T3-L1 adipocytes differentiation (33). Takeda et al. proposed *FA2H* as a novel gene delivering differentiation signals in the breast cancer cell line MDAMB231 (34). These are supportive of our observation that *FA2H* is significantly suppressed in triple negative breast cancer cell lines (Figures 1A–C). In addition, cells lacking *FA2H* expression exhibit increased migration (Figure 4) and CSC proportion (Figure 3). Cells lacking *FA2H* expression are resistant to the synthetic antitumor drug PM02734 (35). As CSCs are known responsible for tumor resistance to drugs and radiation (21, 22), *FA2H* likely plays a tumor suppressive role controlling CSC signalings in breast cancers.

Interestingly, *FA2H* expression is exceptionally high in HCC1937 (Figure 1A). This experimental result is consistent with the results from the 56 cell lines reported by (26), where HCC1937 was classified as a basal-A cell line and showed an exceptional high *FA2H* expression (Figure 1D). However, this exception did not mask the significant difference between luminal and basal (considered TNBC cell lines) cells on *FA2H* expression in their study. Thus, cell lines are not accurate cancer models and may acquire mutations or new molecular features after being cultured *in vitro* for several generations, and dozens of cell lines are needed to exclude the potential violation of one or several exceptional cases on the underlying rules if cell lines were used to examine the roles of genes in subtype differentiation. Also, HCC1937 harbors a *PTEN* deletion (36). It is possible that when *PTEN*, which encodes an important tumor suppressor, is completely lost, other genes with tumor suppressive roles such as *FA2H* are activated as alternative signalings to prevent cells from running chaotic. However, such a hypothesis requires experimental validation and the orchestrated networking needs decryption. It is worth mentioning that being under-expressed in CSCs does not exclude the possibility of *FA2H* being over-represented in HCC1937 cells which is a balanced mixture of CSCs and non-CSCs.

**Figure 7:**
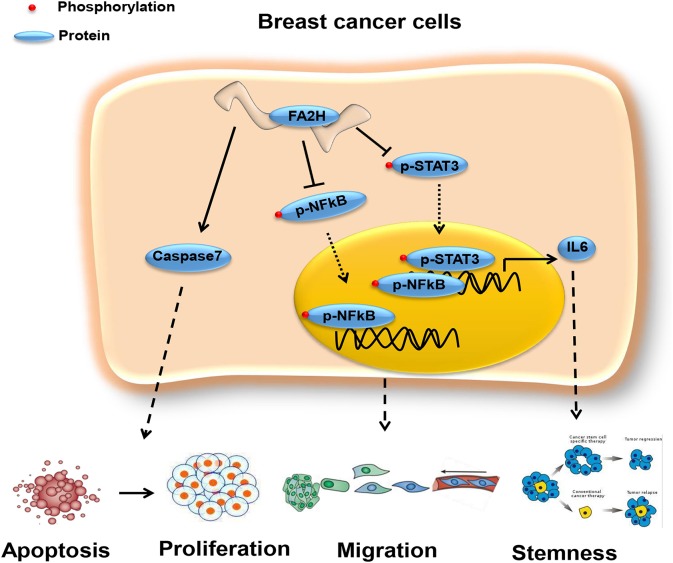
Network revealing the tumor suppressive role of FA2H via the STAT3/IL6 axis and NFkB signaling. FA2H suppresses phosphorylation of NFkB and STAT3, reduces their nuclear transportation that ultimately leads to reduced expression of genes promoting cancer cell migration and stemness. FA2H induces Caspase 7, an executioner of apoptosis, and thus increased apoptosis and reduced proliferation.

Cytokines are known to mediate CSC signalings (24, 37–39). Blocking the CXCR1/IL8 axis has been suggested to selectively target CSCs in breast cancer (24). IL6 could induce tamoxifen resistance in luminal breast cancer (40), and was implicated in CSC maintenance and progenitor-enriched mammosphere formation (28–30). Importantly, IL6 is one of our candidate genes (Table S5) capturing CSC properties in triple negative breast cancer. We, thus, hypothesize that the CXCR2/IL6 axis plays a synergistic role with FA2H in breast CSC signaling and, possibly and in particular, among triple negative breast cancers. For this, we analyzed IL6 expression and cancer cell stemness under *FA2H* down- and up-regulation. As expected, both IL6 expression and CSC percentage significantly were reversely regulated on *FA2H* modulation (Figure 5). These drive us to further explore the mechanisms under such a synergistic effect. STAT3 signaling was known to regulate embryonic stem cell fate (41) and required for CSC activation in hepatic cancer (42). Recently, STAT3/IL6 was reported to mediate signalings endowing breast cancer cells with stem-like properties (43). The binding sites of STAT3 were found enriched in the promoter region of IL6 as predicted using JASPAR (44, 45) (Table 4), consolidating our hypothesis on the link between STAT3 and IL6. Down-regulating *FA2H* was shown to result in STAT3 phosphorylation at Tyrosine 705 (Figures 5, 6), which activates STAT3 mediated surge of IL6 and ultimately increased CSCs, and vice versa (Figure 5). On the other hand, IL6 could activate STAT3, which was reported to promote the proliferation and metastasis of many cancers (46). Such a feedback loop may contribute to the persistent signalings driving CSC properties (Figure 7).

**Table 4:** STAT3 binding sites at the promoter region of IL6 as predicted using JASPAR.

**Predicted binding site sequence**	**Score**	**Relative score**	**Start**	**End**
CTTTCTGGAAA	10.950	0.930209099089144	839	849
TTGCAAGGAAG	9.096	0.907748715679777	1,455	1,465
TTTCCAAAAAA	7.615	0.88980706097143	927	937
GTGTCAGGAAG	7.113	0.883725554783997	820	830
GTGCAGGAAAT	6.456	0.875766292702036	301	311
GAGCTTGGAAC	5.315	0.86194358640749	1,525	1,535
CTGCTGGAACA	3.273	0.837205666816375	1,333	1,343
TTGCCAGGATG	3.050	0.834504121239088	1,572	1,582
TTTCTTAGAGA	2.820	0.831717773782694	1,604	1,614
TTTCTGGAAAA	13.617	0.962518615029157	840	850
GTTTTTGGAGA	1.320	0.813545942545342	1,466	1,476
TTGGTAGAAAG	1.065	0.810456731234992	1,177	1,187
CTGGCAGAAAA	1.054	0.810323471139251	1,750	1,760
CTGTTTGGTAG	0.617	0.805029410972103	1,173	1,183
ATTCCTCAAAG	0.569	0.804447912372507	1,065	1,075

*Model ID for STAT3 is MA0144.2. Binding sites in the positive strand are recorded*.

Among the three pathways that canonically control cancer cell stemness, migration and proliferation, we found that *FA2H* did not alter the total phosphorylation but the nuclear phosphorylation level of JNK. It has been reported that phosphorylation is a pre-requisite of the degradation of protein MafA under low-glucose conditions (47). Thus, it is possible that phosphorylated JNK is not effectively transferred into nucleus that leads to reduced cell proliferation and seemingly unaffected phosphorylation levels observed; and phosphorylated JNK remains in the cytoplasm to assist in the rapid degradation of its total protein. In addition, we could not exclude the possibility that FA2H affects other proliferation pathways such as WNT signaling besides the MAPK cascade. These unconfirmed or unaddressed questions are left out for future studies.

NFkB is a canonical pathway controlling cell migration, inflammation and has been associated with cancer stem cells (48, 49), and targets many genes associated with cancer progression including IL6 (49). We are thus motivated to explore whether and how NFkB signaling is altered on FA2H perturbation. Decreased NFkB phosphorylation level was observed in triple negative cells with *FA2H* over-expression and vice versa (Figure 6), suggesting that the suppressive role of *FA2H* on IL6 secretion is mediated through both NFkB and STAT3 signalings.

The protein product of *FA2H* is involved in the synthesis of 2-hydroxysphingolipids with comparably less clinical functions being studied. We report, in this study, its novel tumor suppressive roles via CSC control on breast cancer cells and the STAT3/IL6 axis and NFkB signaling that drive this functionality. Other pivotal genes and pathways deciphered in this study on the maintenance of CSCs worth in-depth investigations for diagnostic and therapeutic purpose against triple negative breast cancers and warrant further studies.

## Data Availability Statement

The transcriptomic data generated from this study is deposited in Gene Expression Omnibus (GEO) as GSE132083.

## Ethics Statement

All authors agree with the content of this paper and give consent for its publication. Human primary breast cancer tissues used in this study were collected from the Affiliated Hospital of Jiangnan University on approval of the Clinical Research Ethics Committees of Affiliated Hospital of Jiangnan University.

## Author Contributions

XD designed and supervised the study, conducted the bioinformatics analysis, drafted the paper, and financially supported this project. SZ and HC conducted most experiments. DC collected clinical samples and conducted immunohistochemistry staining. XC helped in statistics calculations. ZH constructed the stable cell lines for *FA2H* modulation.

### Conflict of Interest

The authors declare that the research was conducted in the absence of any commercial or financial relationships that could be construed as a potential conflict of interest.

## Abbreviations

CSC: cancer stem cell
TNBC: triple negative breast cancer
ER: estrogen receptor
PR: progesterone receptor
HER2: epidermal growth factor receptor 2
FPKM: fragments per kilobase million reads.
