# Supplementary figures and images for: FA2H Exhibits Tumor Suppressive Roles on Breast Cancers via Cancer Stemness Control
Source: Front Oncol. 2019 Oct 24;9:1089. doi: 10.3389/fonc.2019.01089 (PMC6821679; doi:10.3389/fonc.2019.01089)

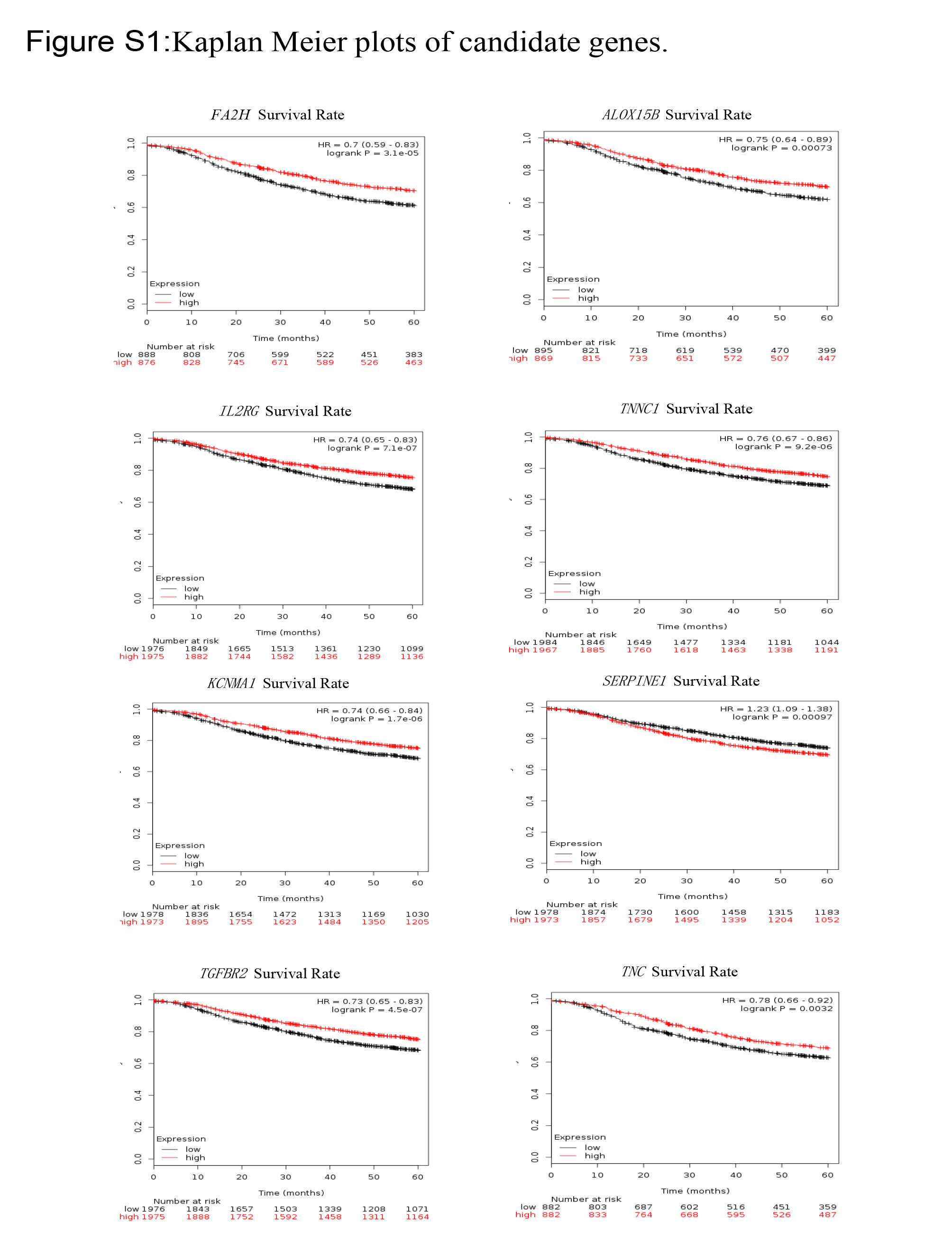

Supplement: Supplementary file 2 [file Image_1.TIF]

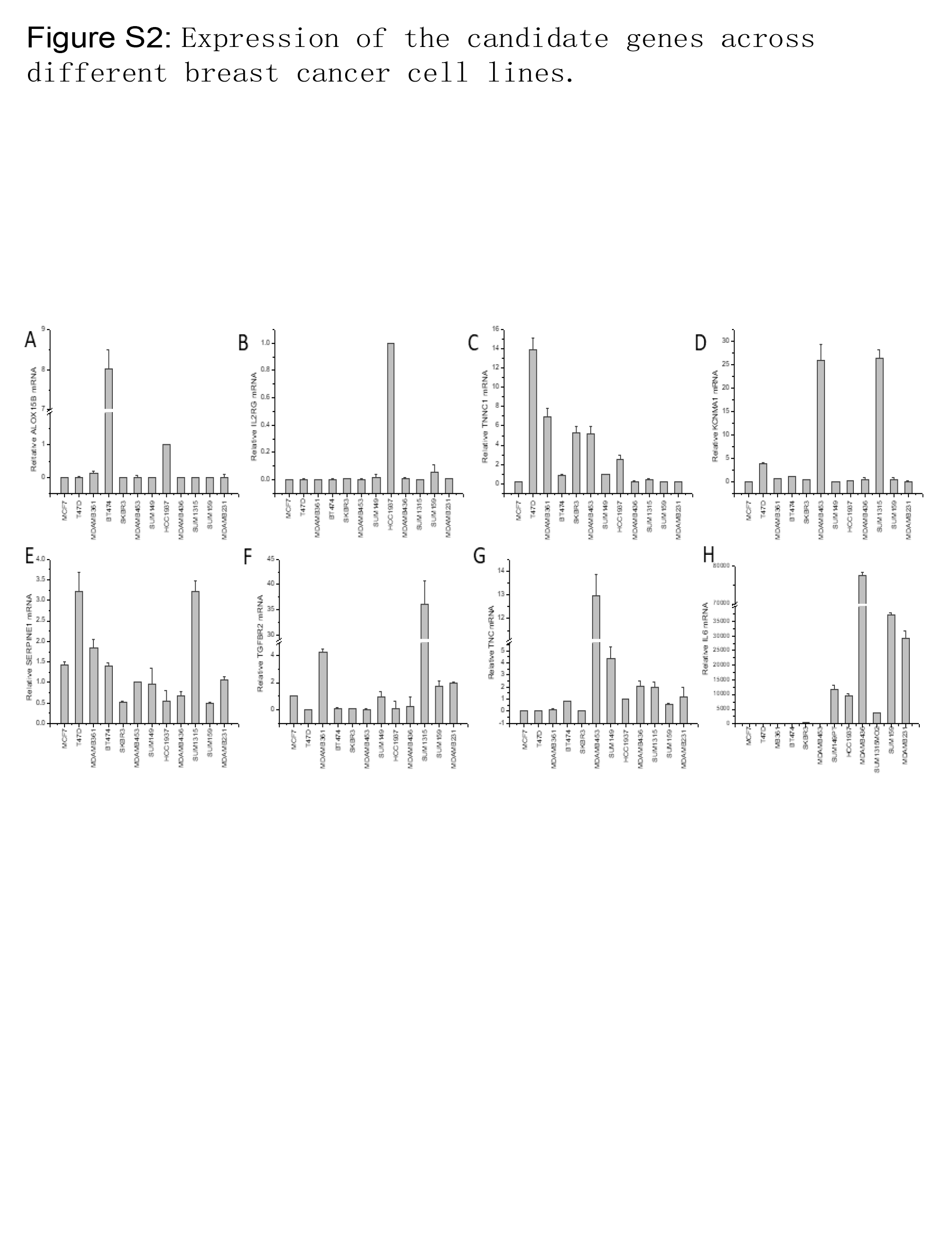

Supplement: Supplementary file 3 [file Image_2.TIF]

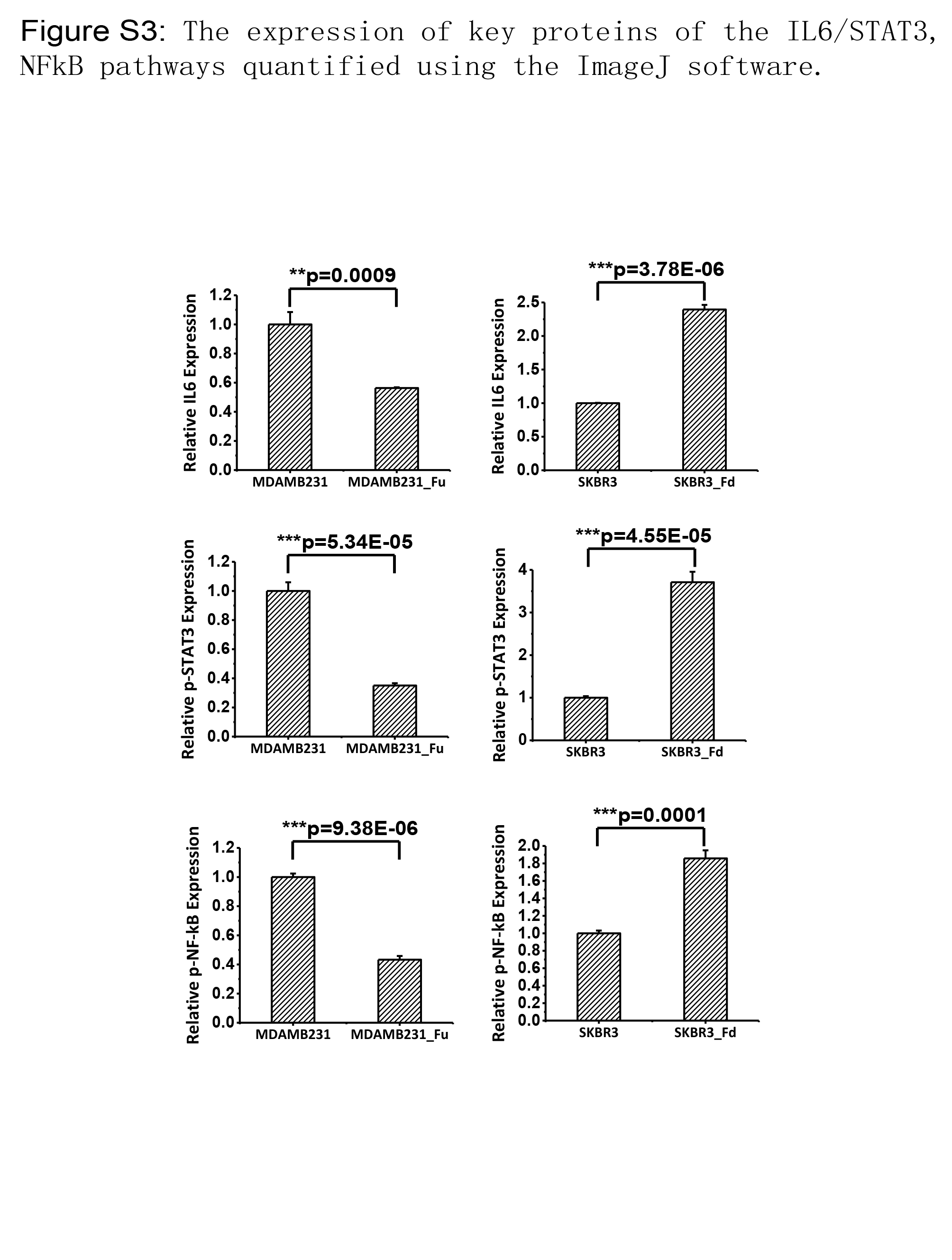

Supplement: Supplementary file 4 [file Image_3.TIF]

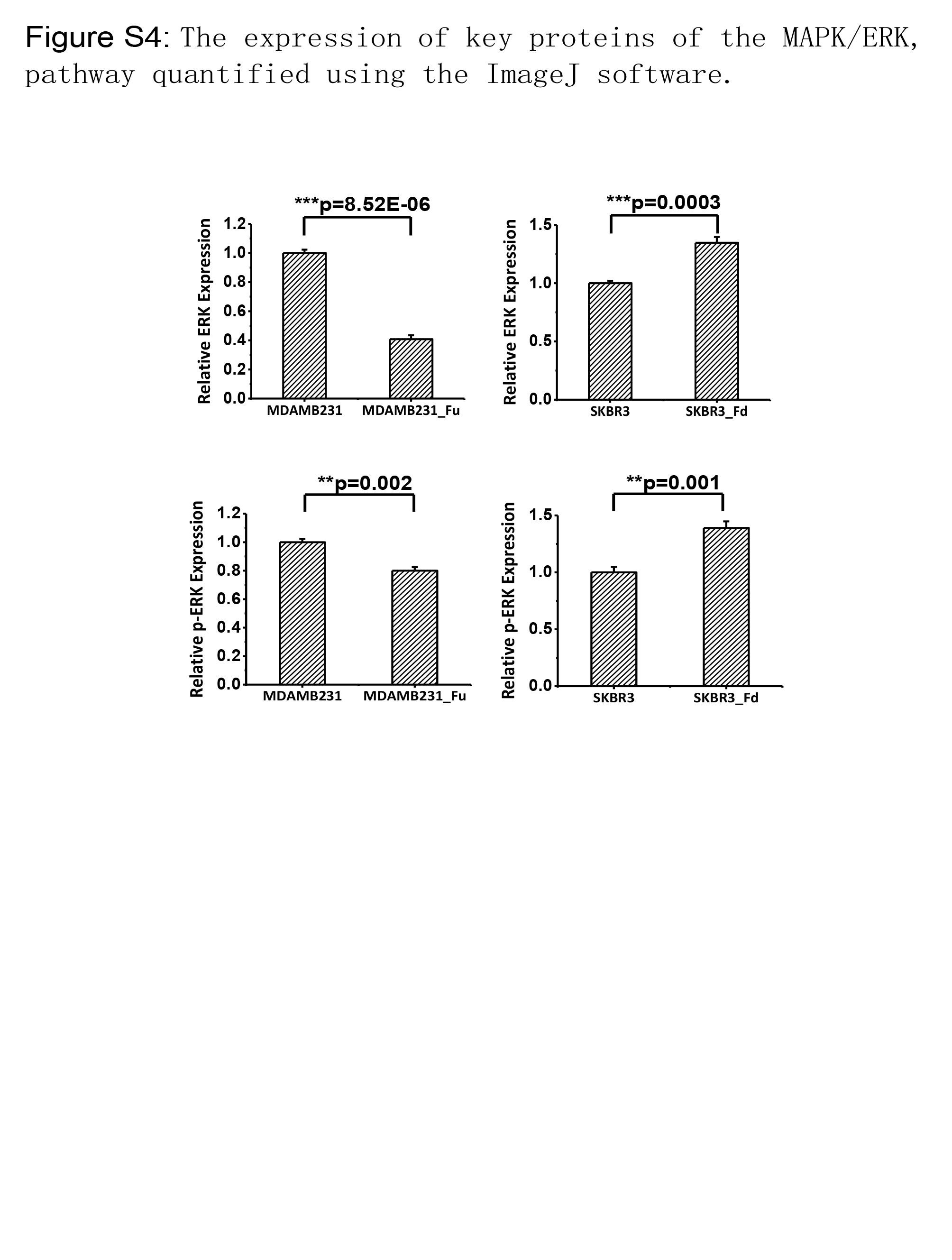

Supplement: Supplementary file 5 [file Image_4.TIF]

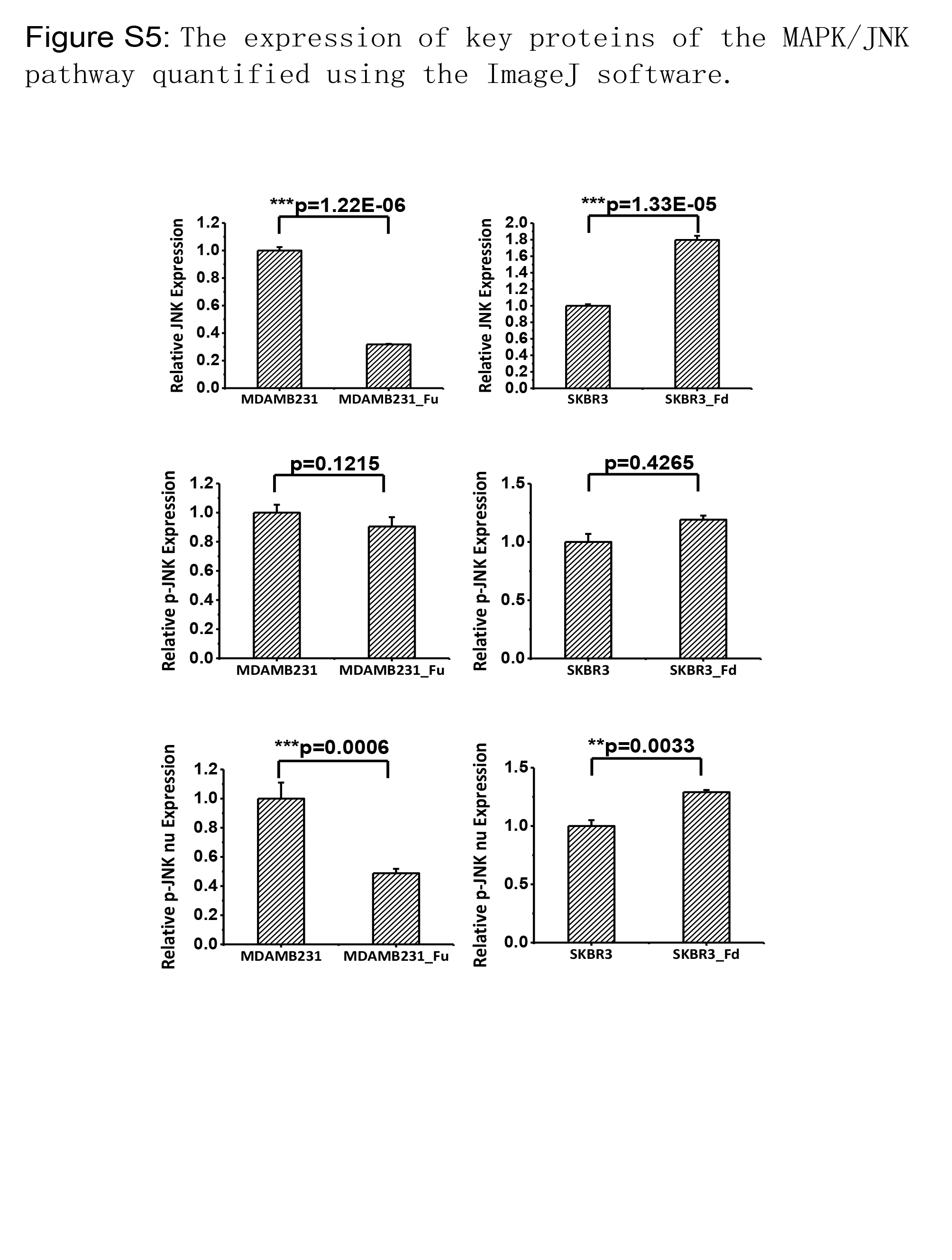

Supplement: Supplementary file 6 [file Image_5.TIF]
